# Study on the Mechanical Properties, Tensile Performance, Hydration Heat, and Microstructure of VAE-Modified Rubber Mortar

**DOI:** 10.3390/ma18030651

**Published:** 2025-02-01

**Authors:** Jiaming Zhang, Ce Bian, Bowen Chen, Chunhe Li, Hua Wei, Hao Lu

**Affiliations:** 1 Materials & Structural Engineering Department, Nanjing Hydraulic Research Institutes, Nanjing 210029, China; jiamingz0110@hhu.edu.cn (J.Z.); luhaoxiaobai@163.com (H.L.); 2 College of Water Conservancy and Hydropower Engineering, Hohai University, Nanjing 210098, China; 3 General Institute of Water Resources and Hydropower Planning and Design, Ministry of Water Resources, Beijing 100120, China; biance123123@126.com; 4 Xinjiang Shuifa Power Energy Group Co., Ltd., Urumqi 830009, China; bowenchen615@163.com; 5 Department of Civil and Environment Engineering, University of Miyazaki, Miyazaki 889-2192, Japan

**Keywords:** tensile strength, ultimate tensile strain, hydration heat, early-age crack resistance, waste rubber powder, vinyl acetate-ethylene, cement-based materials

## Abstract

This study builds on the practice of using waste rubber to improve the ductility of cement mortar and further explores the potential of vinyl acetate-ethylene (VAE) in enhancing the ductility of rubber cement mortar (RM). It systematically analyzes the effects of VAE on the workability, mechanical properties, crack resistance, and microstructure of RM. Additionally, isothermal calorimetry was employed to investigate the mechanism of VAE’s influence on cement hydration heat. The results show that VAE significantly improves the flexural strength, tensile strength, and ultimate tensile strain of RM, while reducing its compressive strength and tensile elastic modulus, thereby markedly enhancing its flexibility and ductility. At a VAE content of 4%, the fluidity, 28-day flexural strength, tensile strength, and ultimate tensile strain of RM reached 240 mm, 4.83 MPa, 1.92 MPa, and 233 × 10^−6^, respectively, representing increases of 16%, 18.97%, 11.63%, and 62.94% compared to ordinary RM. However, when the VAE content exceeded 4%, both flexural strength and tensile strength began to decrease. Furthermore, the incorporation of VAE induced the formation of flexible polymer films within the RM matrix but also increased the porosity of the cement matrix, extended the induction period of cement hydration, and reduced the rate and degree of hydration. These findings provide valuable data to support the development of high-ductility and high-crack-resistance concrete repair materials.

## 1. Introduction

Waste tires are a significant type of solid industrial waste. With the development of the rubber and automotive industries, the accumulation of waste tires, rubber products, and trimmings has increased substantially [1]. In 2024, China’s production of waste tires is projected to reach approximately 330 million, weighing over 10 million tons, with the annual generation from scrapped tires being expected to grow at a rate of 6% to 8%. The traditional disposal methods for waste rubber tires typically include landfilling and incineration. However, due to the low density and large volume of rubber, landfilling requires significant land resources, and the natural degradation of rubber takes a long time. The degradation may also cause surface and groundwater pollution, destruction of vegetation, threat to human health and other safety hazards [2]. Additionally, the incineration of rubber generates a large number of toxic fumes, bringing serious air pollution [3] and posing fire risks. Therefore, there is an urgent need to explore new treatment methods for used rubber.

In recent years, researchers have introduced waste rubber into cement-based materials and discovered its potential to improve the toughness and durability of these materials. Mehmet’s study showed that incorporating 10% rubber increased the fracture energy of pervious concrete by 28% [4]. Kanokon further demonstrated that waste tire rubber, by filling the voids in fiber-reinforced cement-based composites, enhanced their flexural strength [5]. Similarly, Peng et al. [6] conducted indirect tensile strength tests on cement-stabilized aggregate (CSA) with varying rubber content, showing that the ultimate failure strain increased by 20.3% when the rubber content reached 28.6%. From a microstructural perspective, Ahmed Hilal Farhan [7] used X-ray CT to observe that rubber particles embedded in a rigid matrix extended crack propagation paths, thereby increasing energy dissipation and enhancing ductility and energy absorption capacity. Furthermore, Fatema [8] found that the wear resistance of rubber-modified cement mortar improved by 17% at a 7% rubber content due to optimal particle distribution. However, the low thermal conductivity, smooth surface, and the presence of zinc stearate in tire formulations weaken the interfacial bonding between rubber and cement, thereby reducing the compressive strength, elastic modulus, and workability [9]. Su’s research [10] indicated that concrete slump decreased regardless of rubber particle size due to the strong water absorption capacity of rubber, which reduced the free water in the mixture. Ashwin [11] reported that when the rubber content was 10%, 20%, and 30%, the compressive strength of geopolymer composites decreased by 12%, 21.74%, and 34.33%, respectively.

At the same time, organic polymers have proven effective in improving the flexibility and tensile performance [12] of cement-based materials while enhancing the workability of cement paste, thereby facilitating construction [13]. Shaise K. et al. [14] found that incorporating 6% SBR latex into geopolymer mortar led to 17.4% and 6.8% increases in splitting tensile strength and flexural strength, respectively, with minimal changes in compressive strength. Additionally, the formation of a sealing film reduced drying shrinkage. Pan [15] demonstrated that SA latex improved the workability of fresh mortar and enhanced flexural and bonding strengths through interactions with hydration products, although its effects diminished beyond an 8% content. SEM analysis showed that while PA did not form a continuous polymer film within the mortar, it still improved flexural strength by limiting the initiation and propagation of microcracks [16]. Among various organic polymers, VAE has gained attention for its unique mechanism. In the highly alkaline environment of cement paste, VAE undergoes hydrolysis and reacts with Ca^2+^ to form a continuous flexible network film [17]. This structure exhibits excellent deformation compatibility, effectively enhancing the toughness [18], flexural strength, and stress absorption capacity [19] of mortar and concrete. Additionally, the protective colloid polyvinyl alcohol (PVOH) on the VAE surface efficiently disperses particles in the paste, preventing particle agglomeration and improving workability through a ball-bearing effect [20].

Despite significant advances in mortar modification, few studies have explored the combined effects of rubber powder and VAE on the flexibility and ductility of mortar, particularly in engineering applications requiring enhanced deformation adaptability. Most existing research has focused on either rubber-modified mortars or VAE-modified mortars separately, leaving a gap in understanding their synergistic effects. Therefore, this study aims to explore the potential of further enhancing the flexibility and ductility of mortar by introducing VAE based on the incorporation of an appropriate amount of rubber powder into ordinary cement mortar. The findings provide data support and practical guidance for engineering applications, such as PCCP pipe joints, large-area concrete slab surface repairs, and joint treatments, which have lower strength requirements but higher demands for deformation adaptability.

## 2. Materials and Methods

### 2.1. Materials

The cement used in the test was Conch P-O 42.5 ordinary silicate cement, conforming to the Chinese standard GB175-2023 [21]. The main chemical composition was tested using XRF (X-ray fluorescence spectroscopy), as shown in Table 1. The fineness modulus is an index used to measure the particle size distribution of sand. The larger the value of the fineness modulus, the coarser the sand particles. According to GB/T 14684-2022 [22], the fineness modulus of the river sand used in this experiment was 2.68, with an apparent density of 2600 kg/m^3^. The rubber powder was sourced from waste tires in Hunan Province, China, with a maximum size of 425 μm. The VAE re-dispersible latex powder was produced by Sanwei Company in Shanxi, Linfen, China. According to GB/T 29594-2013 [23], its bulk density was 464 g/L, its average particle size was 84 μm, and its glass transition temperature was 13 °C. The mixing water was tap water.

### 2.2. Test Program

This experiment employed a single-factor test method to study the effect of VAE on the workability, mechanical properties, tensile properties, toughness, brittleness, and heat of hydration of RM. According to the existing research results of the group, when the volume ratio of rubber powder to sand is 3:1, RM has the best flexibility and tensile properties, and the magnitude of the loss of fluidity and mechanical properties is low [24,25]. Therefore, in this test, the volume ratio of rubber powder to sand was fixed at 3:1 (corresponding to a mass ratio of 1.245:1). Other ratios included a binder–sand ratio of 2.5:1 (by weight), a water-cement ratio of 0.38 (by weight), and VAE contents of 0%, 2%, 4%, 6%, and 8% by cement weight, resulting in 5 groups of tests, with the 0% VAE group serving as the control. The specific mixing ratios are shown in Table 2.

The preparation process for VAE-modified RM involved mixing cement, sand, rubber powder, and VAE for 30 s, followed by the addition of water and low-speed mixing for 60 s. This was then followed by high-speed mixing for 30 s to obtain the VAE-modified RM. Finally, the mixture was filled into molds and vibrated for 90 s. After resting for 24 h at an ambient temperature of 20 °C, the specimens were demolded and cured in a standard curing room at 20 °C and 95% relative humidity until the specified curing age.

### 2.3. Test Methods

Table 3 presents the performance test types, sample dimensions, number of samples, and the relevant standards followed for each experimental group in both fresh and hardened states.

#### 2.3.1. Fluidity Test

**Figure 1:**
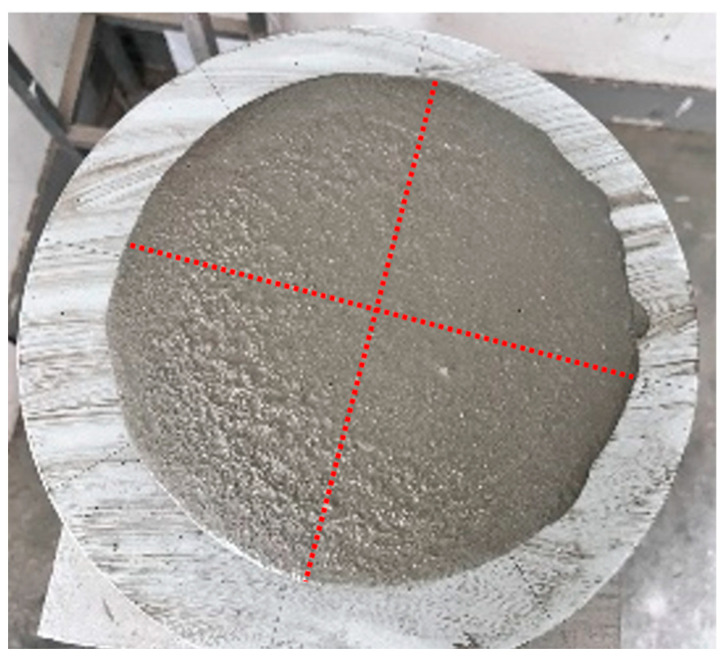
Mortar fluidity test.

The fluidity test method follows GB/T2419-2005 [26], as shown in Figure 1. Prior to the fluidity test, the jumping table, mold, and other equipment needed to be wetted. Once the mortar was mixed, the mold was placed at the center of the jumping table. The mortar was added in two stages: the first stage filled the mold to two-thirds of its height, and the mortar was compacted evenly with 15 taps; the second stage filled the mold until the mortar rose 20 mm above the top of the mold, and it was compacted evenly with 10 taps. Afterward, the surface of the mortar was leveled, and the mold was gently lifted vertically. The jumping table was then immediately activated, completing 25 jumps at a rate of one per second. The diameters of the mortar base in two mutually perpendicular directions were measured with a caliper, and the average value was calculated as the final result.

#### 2.3.2. Mechanical Strength Test

**Figure 2:**
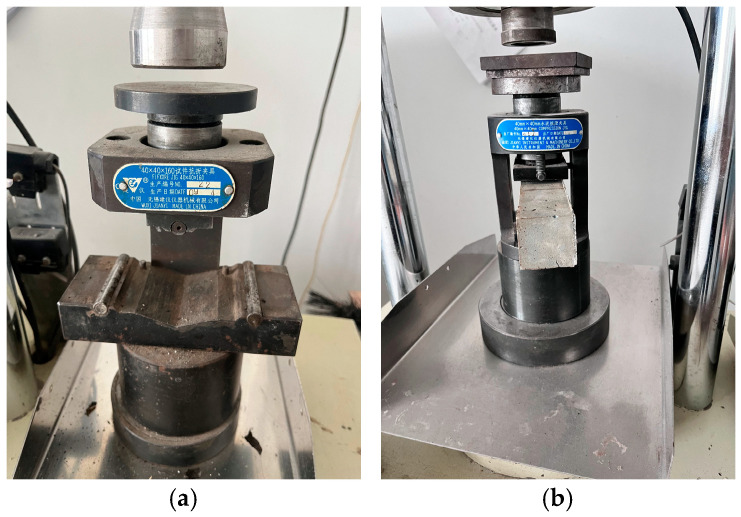
Flexural strength and compressive strength tests. (**a**) Flexural strength test; (**b**) compressive strength test. The non-English terms in (**a**,**b**) pertained to the serial numbers, manufacturing dates, and manufacturers of the flexural test jigs and compression test jigs, respectively.

Flexural strength and compressive strength tests of cement mortar were conducted according to the GBT/17671-2021 standard [27]. The testing equipment model was TYE-300D (WUXI JIANYI company, Wuxi, China), with a specimen size of 40 mm × 40 mm × 160 mm, as shown in Figure 2. The curing ages were 7 days and 28 days.

During the flexural strength test, the load was applied vertically on the opposite sides of the prism until fracture occurred. The loading rate was controlled within the range of 50 N/s ± 10 N/s. The flexural strength was calculated according to Formula (1), with three specimens tested for each mix ratio, and the average of the measured values was taken as the final result.

(1) $$R_{f}=\frac{1.5F_{f}L}{b^{3}}$$

where R_f_ is the flexural strength (MPa); F_f_ is the load applied to the middle of the prism at fracture (N); L is the distance between the support cylinders (mm); b is the edge length of the square cross-section of the prism (mm).

After the flexural strength test, the fractured halves of the specimens were used for the compressive strength test. The load was applied uniformly to the side surface of the half-prism until failure, with the loading rate controlled within the range of 2400 N/s ± 200 N/s. The compressive strength was calculated according to Formula (2), with six specimens tested for each mix ratio, and the average value of the measured results was taken as the final result.

(2) $$R_{c}=\frac{F_{c}}{A}$$

where R_c_ is the compressive strength (MPa); F_c_ is the maximum load applied at failure (N); L is the distance between the support cylinders (mm); A is the compressive area (mm^2^).

**Figure 3:**
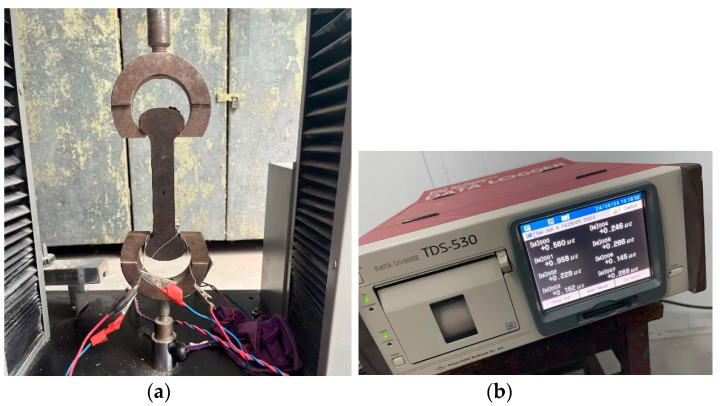
Axial tensile test. (**a**) Dumbbell-shaped axial tensile specimens; (**b**) TDS-530 Static Data Collector.

The axial tensile test was conducted according to the SL352-2020 standard [28]. The test parameters included the ultimate tensile strain, axial tensile strength and tensile elastic modulus of the mortar. The ultimate tensile strain refers to the strain value when the material reaches the maximum tensile force during stretching. It is an important indicator for evaluating the ductility and toughness of a material. In brittle materials, the ultimate tensile strain is often smaller. The tensile modulus of elasticity, also known as the tensile modulus, is a physical quantity that describes a material’s ability to resist elastic deformation under tensile force. Within a material’s elastic range, the larger the tensile modulus of elasticity, the more difficult it is for the material to stretch, indicating higher rigidity. The axial tensile specimens were dumbbell-shaped, as shown in Figure 3a. There were 6 specimens for each mix ratio, and the average value was taken as the final test result. The strain collector used for the test was a TDS-530 static data collector (Tokyo Measuring Instruments Laboratory Co., Ltd., Tokyo, Japan), as shown in Figure 3b. The tensile loading rate was controlled at 0.24 MPa/min.

The axial tensile strength was calculated according to Formula (3).

(3) $$f_{t}=\frac{P}{A}\times 1000$$

where f_t_ is the axial tensile strength (MPa); P is the destructive load (kN); A is the cross-sectional area of the specimen (mm^2^).

According to Formula (3) for calculating the stress σ at different loads, the stress–strain curve for the whole tensile test process was plotted. Then, the curve was fitted with a second-order polynomial, and the destructive stress was substituted to obtain the strain value, which represented the ultimate tensile strain of the specimen.

The axial tensile modulus of elasticity was defined as the secant modulus of elasticity at 0–40% of the destructive stress, and was calculated in accordance with Formula (4).

(4) $$E_{t}=\frac{\sigma_{0.4}}{\varepsilon_{0.4}}$$

where E_t_ is the axial tensile modulus of elasticity (GPa), σ_0.4_ is the 40% breaking stress (MPa), and ε_0.4_ is the strain corresponding to the 40% breaking stress.

#### 2.3.3. Early-Age Crack Resistance Test

**Figure 4:**
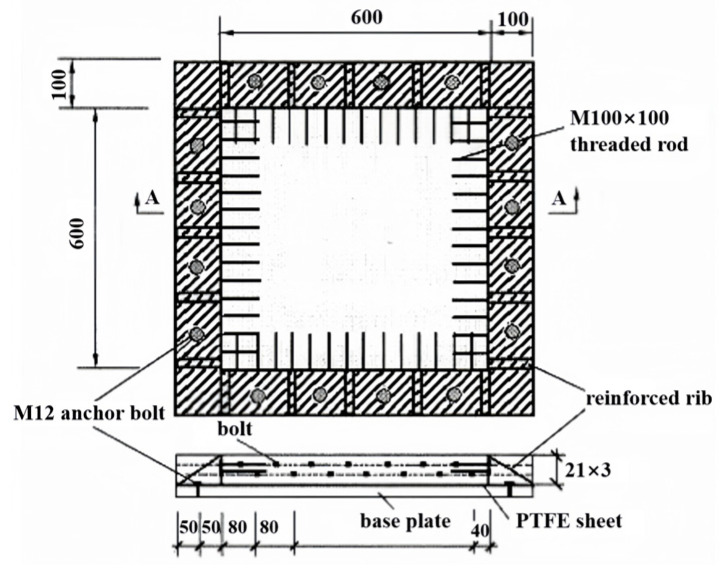
Early-age crack resistance specimen mold for concrete.

The early-age crack resistance of cement mortar under restrained conditions was tested following the Chinese JTG 3420-2020 Standard [29]. Testing was conducted using flat slab specimens with dimensions of 600 mm × 600 mm × 63 mm, as shown in Figure 4. The procedure was as follows: after casting the mortar into the mold and smoothing the specimen surface, it was immediately covered with plastic film. Two hours later, the plastic film was removed, and the fan position and speed were adjusted to achieve an air velocity of 5 m/s ± 0.5 m/s at a height of 100 mm directly above the center of the specimen surface. After 24 h, a steel ruler and crack width gauge were used to measure and record the length and maximum width of each crack.

The evaluation criteria for early-age crack resistance include four specific standards: (1) only very fine cracks are present; (2) the average crack area is less than 10 mm^2^; (3) the number of cracks per unit area is fewer than 10 cracks/m^2^; (4) the total crack area per unit surface area is less than 100 mm^2^/m^2^. Based on these criteria, crack resistance was categorized into five levels as shown in Table 4. Two specimens were tested for each mix proportion, and the average result was taken as the final test value.

#### 2.3.4. Water Permeability Test

**Figure 5:**
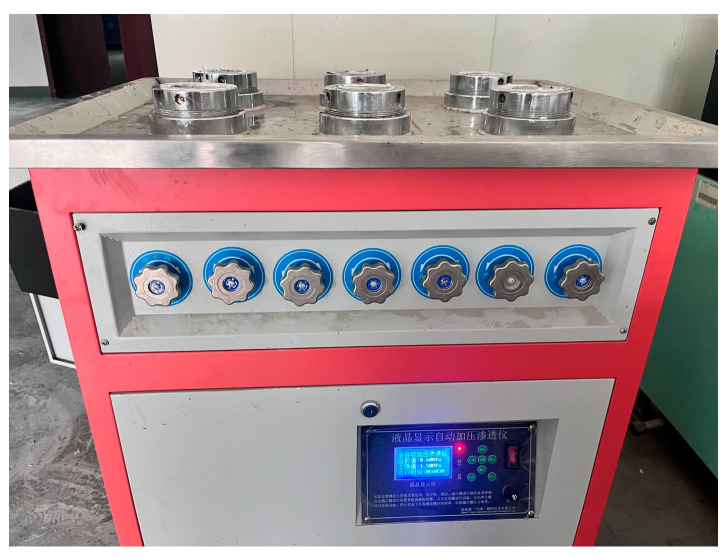
Automatic pressure permeability apparatus. The non-English terms pertained to the operational precautions for the testing apparatus.

The water permeability of RM with different VAE contents was tested according to SL352-2020 [28]. A truncated cone-shaped metal mold was used, with upper and lower diameters of 70 mm and 80 mm, respectively, and a height of 30 mm. At 28 days of curing, the sides of the specimens and the inner surface of the mold were sealed with silicone rubber. After 24 h of standing, the specimens were placed into the permeameter shown in Figure 5. The water pressure was gradually increased to 1.5 MPa and maintained for 1 h. After this period, the surface of the specimen was inspected for leakage. If no leakage occurred, the specimen was split to measure the water penetration height. Three specimens were tested for each mix ratio, and the average value was taken as the final experimental result.

#### 2.3.5. Hydration Heat Test

Hydration heat tests were conducted using a TAM AIR eight-channel isothermal calorimeter on cements with VAE contents of 0%, 2%, 4% and 8%. The solid weight of each test sample was 5 g, and the water-cement ratio was kept consistent with that of the mechanical performance test group, i.e., 0.38. The mix ratios for each test group are shown in Table 5.

#### 2.3.6. Microstructure Analysis

The microstructure of rubber mortar samples of VAE-0, VAE-4 and VAE-8 was observed and analyzed at a curing age of 28 days using scanning electron microscopy (SEM). The samples were extracted from the interior of 28-day specimens after compressive strength tests. To enhance the conductivity and imaging quality, the sample surfaces were treated with high-vacuum gold sputtering. Subsequently, the specimens were mounted on a sample holder and analyzed for microstructural features using SEM.

## 3. Results and Discussion

### 3.1. Fluidity

**Figure 6:**
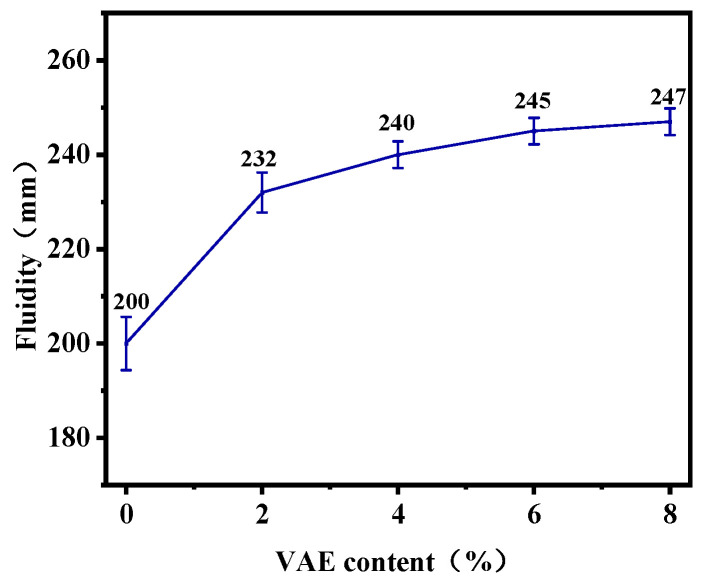
Effect of VAE on the fluidity of RM.

The fluidity of mortar is a key indicator for evaluating its workability [30]. The results of the RM fluidity test with different VAE contents are given in Figure 6. It can be seen that the fluidity of RM increases gradually with the increasing VAE content. Compared to VAE-0, the fluidity of VAE-2, VAE-4, VAE-6 and VAE-8 increased by 16%, 20%, 22.5% and 23.5%, respectively. This indicates that moderate amount of VAE can improve the fluidity of RM. However, the improvement in fluidity gradually diminished with the increase in VAE content.

The enhancement of mortar fluidity by VAE can be attributed to its surface water-soluble protective colloid, polyvinyl alcohol (PVOH), which prevents aggregation between particles [31] and produces a fly ash-like balling effect [32] in the slurry, thereby reducing internal friction, which is manifested macroscopically in the improvement of the fluidity of the mortar. Additionally, VAE exhibits air-entraining properties [33], introducing tiny air bubbles that increase the compressibility of the mortar and further improve its fluidity. However, excessive VAE leads to an increase in the number of solid particles in the slurry, and the average distance between particles decreases. This results in greater interaction force and friction between particles, impeding the ball-bearing effect [20], which is macroscopically manifested as increased viscosity [34] and decreased fluidity of the mortar.

### 3.2. Mechanical Properties

#### 3.2.1. Flexural and Compressive Strength

**Figure 7:**
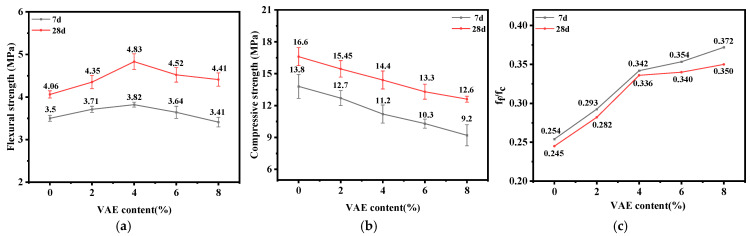
The 7 d and 28 d flexural strength, compressive strength and toughness of VAE-modified RM. (**a**) Flexural strength; (**b**) compressive strength; (**c**) toughness (f_f_/f_c_).

This test evaluated the effects of different VAE contents on the flexural and compressive strengths of RM, and the results are illustrated in Figure 7. The 7 d and 28 d flexural strengths of the RM specimens with different VAE contents are given in Figure 7a, which shows that when the VAE content increased from 0% to 4%, the 7 d and 28 d flexural strengths increased by 9.14% and 18.97%, respectively. However, with further increases in VAE content, the flexural strength began to decrease, though it remained higher than that of the control group; specifically, the 28 d flexural strength of VAE-8 exceeded that of the control group by 8.62%. Thus, the optimal content for flexural strength is 4%.

Figure 7b represents the effect of varying VAE contents on the compressive strength of RM. It can be seen that the 7 d and 28 d compressive strengths of the specimens decreased with the increase in VAE content. Compared to VAE-0, the 28 d compressive strength of VAE-2, VAE-4, VAE-6 and VAE-8 decreased by 6.93%, 13.25%, 19.88%, and 24.1%, respectively. For each additional 1% of VAE, the 28 d compressive strength of RM decreased by about 3.28%.

The ratio of the flexural strength to the compressive strength(f_f_/f_c_) of cement-based composites is a crucial indicator for judging their toughness; the higher this ratio, the better the toughness of the material is considered to be [35]. Ordinary cement mortar is typically a brittle material, with an f_f_/f_c_ ratio generally ranging from 0.083–0.125 [36]. As shown in Figure 7c, the f_f_/f_c_ ratio of VAE-modified RM is between 0.245 and 0.350, which is obviously higher than that of ordinary mortar. This indicates that rubber powder significantly improves the toughness of the mortar, and the toughness is further enhanced through VAE modification. From Figure 7c, it can be observed that with increasing age, the f_f_/f_c_ ratios of RM decrease across all VAE contents, indicating that as hydration progresses, the compressive strength develops faster than the flexural strength. Compared with VAE-0, the f_f_/f_c_ ratio of VAE-2, VAE-4, VAE-6 and VAE-8 increases by 15.10%, 37.14%, 38.78% and 42.86%, respectively. This shows that a low content of VAE increases the f_f_/f_c_ ratio of RM. However, the improvement gradually diminishes as the VAE content continues to increase.

An appropriate amount of VAE can improve the flexural strength and toughness of cement mortar because VAE is hydrolyzed to generate carboxyl groups in the alkaline-rich environment of the cement system. The Ca^2+^ combines with -COO^−^, forming bridges between the polymer chains and creating an interpenetrating flexible polymer--network-like structure inside the mortar. When the mortar is subjected to external forces, the flexible network can effectively absorb and alleviate the internal micro-stresses, which positively contributes to the flexural strength. However, excessive VAE leads to a decrease in both flexural and compressive strength. On the one hand, VAE has an air-entraining effect, and excessive VAE will introduce too many pores [37], resulting in reduced density and mechanical properties of the mortar. On the other hand, VAE can adsorb onto the surfaces of cement particles and hydration products, preventing mineral dissolution and reducing the nucleation sites for C-S-H [38], thereby decreasing the amount of cement hydration products formed, which hinders strength development. Moreover, after the introduction of VAE, both the strength and elastic modulus of VAE itself and the flexible phase that it forms are significantly lower than those of other phases in the cement matrix, weakening the overall strength of the matrix.

#### 3.2.2. Tensile Strength, Ultimate Tensile Strain and Tensile Modulus

**Figure 8:**
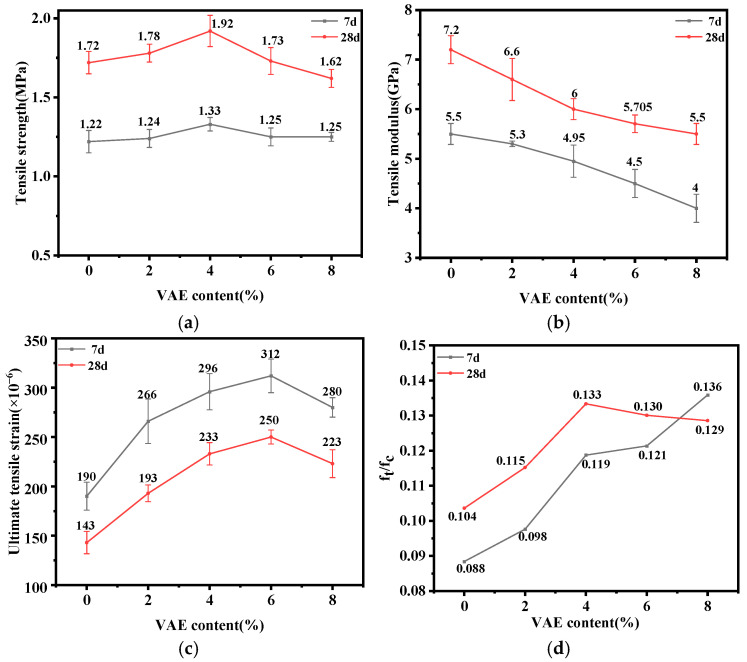
The 7 d and 28 d tensile properties of VAE-modified RM. (**a**) Tensile strength; (**b**) tensile modulus; (**c**) ultimate tensile strain; (**d**) brittleness ratio.

Cement-based materials are typically brittle with poor ductility and tensile properties, making them susceptible to cracking under tensile loads, shrinkage stresses, temperature stresses, and impact loads. Key parameters reflecting the ductility and tensile properties of mortar include axial tensile strength, tensile elastic modulus, and ultimate tensile strain [39]. Figure 8a–d show the effects of different VAE contents on the axial tensile strength, ultimate tensile strain, tensile elastic modulus, and brittleness ratio of RM, respectively. It can be seen that the axial tensile strength and tensile elastic modulus of RM increase with curing age, while the ultimate tensile strain decreases across all VAE contents. This trend can be attributed to the ongoing hydration process, which densifies the internal structure of the mortar, increasing its rigidity and brittleness but reducing ductility and flexibility. In addition, the flexible polymer film formed by VAE inside the mortar gradually ages, becoming more brittle over time.

As can be seen from Figure 8a, the 7 d and 28 d axial tensile strengths of the specimens initially increase and then decrease with the increase in VAE content. The 28 d axial tensile strength of RM reached the maximum value of 1.92 MPa at the VAE content of 4%, which was an increase of 11.6% compared with that of VAE-0. However, with a further increase in the VAE content to 8%, the 28 d tensile strength was reduced to 1.62 MPa, which was 5.81% lower than the control. This suggests that 4% is the optimal VAE content for axial tensile strength.

Figure 8b shows a decreasing trend in the tensile elastic modulus of RM with the increase in VAE content. Compared to VAE-0, the 28 d tensile elastic modulus of the test group of VAE-2, VAE-4, VAE-6 and VAE-8 decreased by 8.33%, 16.67%, 20.76% and 23.61%, respectively, indicating a diminishing effect on tensile elastic modulus reduction as the VAE content increases.

From Figure 8c, it can be seen that when the VAE content increases from 0% to 6%, the ultimate tensile strain rises from 143 × 10^−6^ to 250 × 10^−6^, a significant increase of 74.83%. However, when continuing to increase the VAE content to 8%, the ultimate tensile strain slightly decreases, but it remains 55.94% higher than the control without VAE. The combined results of tensile strength and tensile elastic modulus indicate that although excessive VAE decreases the tensile modulus and improves the ductility of RM, it also significantly reduces the tensile strength, leading to the destruction of RM under relatively low tensile loads. Therefore, excessive VAE would not result in a continued increase in the ultimate tensile strain.

The brittleness ratio of cement-based materials indicates the ratio of tensile strength to compressive strength; the lower the brittleness ratio, the lower the material brittleness and the better the resistance to cracking [39]. The effects of different VAE contents on the 7 d and 28 d brittleness ratios of RM are presented in Figure 8d. It can be seen that the 7 d brittleness ratio of RM increases with higher VAE content, suggesting that VAE reduces the brittleness of RM in its early stage. At 28 d, the brittleness ratio increases by 28.67% as the VAE content rises from 0% to 4%. Further increasing the VAE content to 8% results in a slight decrease in the brittleness ratio, though it remains 24.13% higher than the control group. The decrease in brittleness ratio may be due to the excess VAE inhibiting the hydration reaction, thus significantly impacting the development of tensile strength.

The decrease in the tensile elastic modulus and increase in the ultimate tensile strain are macroscopically manifested as reduced brittleness in mortar, with enhanced ductility and deformation adaptability. VAE improves the ductility and flexibility of mortar, on the one hand, because of the chemical interactions between VAE and the metal cations, such as Ca^2+^ and Al^3+^, produced by cement hydration, to form flexible polymer network structures. These network structures act like microfibers with a high ultimate strain and low elastic modulus [40], allowing them to elongate, bend, and rotate under a tensile load, thus increasing the toughness and deformation capacity of the mortar [41]. On the other hand, VAE suppresses cement hydration reactions [37], reducing the formation of rigid hydration products responsible for structural stiffness, thereby lowering the overall rigidity and brittleness of the mortar.

### 3.3. Early-Age Crack Resistance

**Figure 9:**
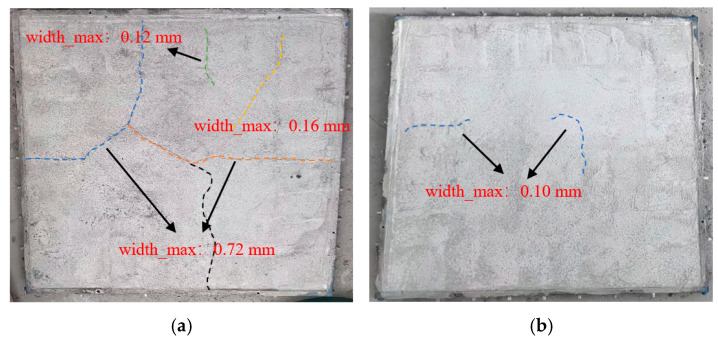
Cracking patterns of RM plates with different VAE contents: (**a**) 0% VAE content; (**b**) 4% VAE content.

The results of the mechanical performance tests in Section 3.2 indicate that at a VAE content of 4%, the VAE-modified RM exhibits optimal 28 d flexural and tensile strengths, as well as a high ultimate tensile strain. These properties are key considerations in engineering applications such as PCCP pipe joints and the repair of large flat concrete structures. To comprehensively evaluate the engineering applicability of VAE-modified RM, this study tested the early-age crack resistance of RM specimens with 0% and 4% VAE content. Figure 9 shows the cracking patterns of VAE-0 and VAE-4, with the crack development direction marked by dashed lines. As shown in Figure 8, the control group mortar exhibited relatively large crack widths, with an average crack density of 11.1 cracks per m^2^, an average crack area of 118 mm^2^ per crack, and a total crack area of 1310 mm^2^/m^2^, which did not meet any of the early-age crack resistance criteria. This indicates a high early-age cracking sensitivity, classifying the control mortar as crack resistance grade V. In contrast, after the addition of 4% VAE, the early-age crack resistance of RM was significantly improved. In VAE-modified RM, the cracks were finer and shorter, with a crack density of 9.9 cracks per m^2^, a reduction of 10.81%, and the average crack area was reduced to 6 mm^2^ per crack, with a total crack area of 59 mm^2^/m^2^. This indicates that the addition of VAE reduced both the number and area of cracks, improving the overall crack resistance of RM. Compared to the control group, the early-age cracking sensitivity of RM was markedly reduced, meeting all four early-age crack resistance criteria, and achieving crack resistance grade I.

The enhancement of the early-age crack resistance with the addition of VAE can be attributed to the formation of a flexible polymer phase within the cement matrix. This polymer phase not only disperses the tensile stresses generated during hardening but also effectively mitigates stress concentration, thus reducing crack initiation and propagation. Additionally, VAE enhances the toughness and interfacial bonding strength of the mortar, effectively in the interface region between the cement matrix and the polymer, which effectively inhibits the propagation of micro-cracks during the hardening process [18]. As cracks become finer and fewer in number, the overall structure of the mortar exhibits better crack resistance,

### 3.4. Water Permeability

**Figure 10:**
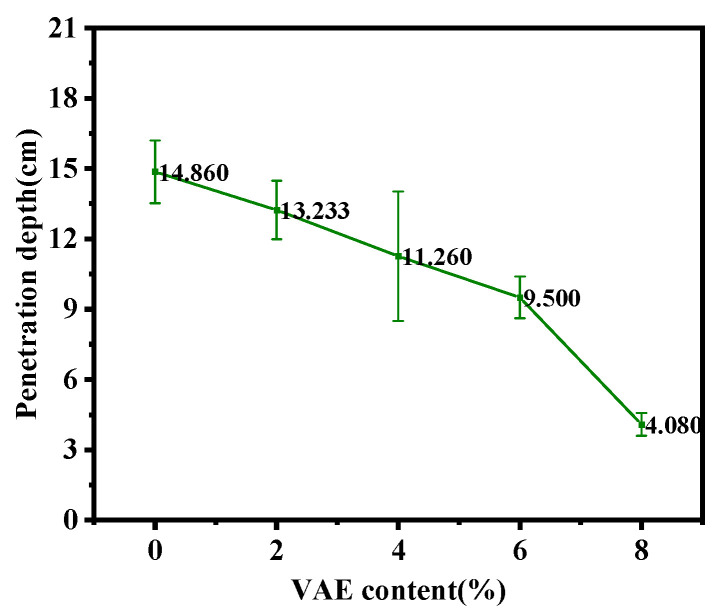
Water penetration depth of RM with different VAE contents.

This study investigated the effects of different VAE contents of 0%, 2%, 4%, 6%, and 8% on the water permeability of RM. The results show that when the water pressure was gradually increased to 1.5 MPa, water did not penetrate the surface of any of the specimens’, demonstrating that all samples exhibited excellent impermeability at this water pressure. To further analyze the depth of water penetration, the specimens were split and the results are shown in Figure 10. It can be observed that as the VAE content increased, the water penetration depth decreased. Compared to VAE-0, the water penetration depth of VAE-2, VAE-4, VAE-6 and VAE-8 decreased by 10.97%, 24.23%, 36.07%, and 72.54%, respectively. This trend indicates that VAE effectively reduces the water penetration depth and improves the water impermeability of RM. Furthermore, the improvement in impermeability becomes more significant with increasing VAE content. This phenomenon can be explained by several factors. First, after the incorporation of VAE, a polymer film forms within the mortar, which acts as a “barrier” in the microstructure, effectively slowing down the water penetration process. This film reduces the pathways for water to penetrate, preventing water from easily penetrating deeper into the matrix through pores or cracks. In addition, VAE enhances the crack resistance of the mortar, making it less likely to develop cracks under water pressure [19]. Since cracks are the primary pathways for water penetration, reducing crack formation directly decreases the pathways for water to permeate, thereby improving the water impermeability of RM.

### 3.5. Hydration Heat

**Figure 11:**
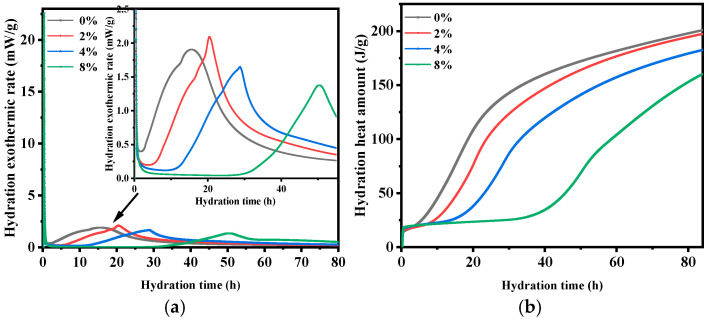
Effect of VAE content on the hydration exothermic rate and hydration heat amount of cement. (**a**) Hydration exothermic rate; (**b**) hydration heat amount. The inset plot in (**a**) represents the heat release during the dissolution process of cement within the first 55 h of hydration.

In order to better reveal the effect of VAE on the cement hydration process, the 84 h hydration heat of cement with different VAE contents was tested through isothermal calorimetry. The VAE-modified RM with 4% VAE content demonstrates significant potential for engineering applications. Moreover, to investigate the effects of no VAE, a low VAE content, and a high VAE content on the heat of hydration, this study selected samples with VAE contents of 0%, 2%, 4%, and 8% for heat of hydration testing, as shown in Figure 11. According to the distinct phases in the heat flow curve, the early hydration process of cement can be divided into five periods: initial fast reaction, induction, acceleration, deceleration and stabilization [37].

From Figure 11a, it can be seen that VAE addition significantly delays cement hydration. As the VAE content increases, the peak time of the initial hydrolysis phase of cement is delayed. Specifically, the peak times for the initial hydrolysis phase of VAE-0, VAE-2, VAE-4, and VAE-8 are 0.179 h, 0.208 h, 0.252 h, and 0.289 h, respectively. The induction period for cement hydration with different VAE contents ends at 2.7 h, 6.9 h, 11.1 h, and 30.3 h, respectively, indicating that VAE markedly extends the induction period, with this effect intensifying as the VAE content increases. During the acceleration phase of hydration, the appearance times for the second exothermic peak in VAE-0, VAE-2, VAE-4, and VAE-8 are 15.521 h, 20.455 h, 28.770 h and 50.365 h, respectively, which shows that VAE delays the peak appearance, and the delay intensifies with increasing VAE content. When the VAE content increases from 0% to 2%, VAE briefly promotes part of the hydration reaction, as reflected in an increase in the second exothermic peak from 1.907 mW/g to 2.094 mW/g. However, with the continued increase in VAE content, the second exothermic peak decreases, with VAE-4 and VAE-8 showing reductions of 13.63% and 27.64%, respectively, compared to VAE-0.

Figure 11b shows the effects of different VAE contents on the total hydration heat of cement. From Figure 11b, it can be seen that the total hydration heat decreases with the increase in VAE content. At any test time, the hydration heat order is: VAE-0 > VAE-2 > VAE-4 > VAE-8. This result indicates that VAE inhibits the hydration reaction, with higher VAE content correlating with a lower hydration degree. The cumulative heat released by different VAE contents in 84 h was 200.99, 197.40, 182.39, and 160.38 J/g, respectively, indicating that low VAE contents minimally impact the extent of hydration, whereas higher contents reduce the hydration degree, which may hinder the long-term strength of cement [17,19], explaining the reduction in compressive strength observed in VAE-modified RM. This occurs because VAE adsorbs onto cement particle surfaces, preventing contact between cement particles and free water [38]. At the same time, the polymer film generated by the reaction between VAE and the cement matrix obstructs the migration of Ca^2+^, SO_4_^2−^, and OH^−^ ions, thereby slowing down the crystallization rate of hydration products, and delaying the overall cement hydration process [42].

### 3.6. Microstructure

**Figure 12:**
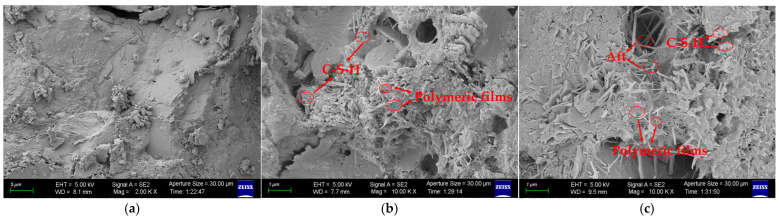
SEM analysis. (**a**) 0% VAE content; (**b**) 4% VAE content; (**c**) 8% VAE content.

To better understand the mechanism by which VAE affects the mechanical properties of RM, we further investigated the microstructural changes in VAE-modified RM using SEM analysis. The 4% VAE exhibited the optimal performance in the key properties of the modified RM. To investigate its impact on the microstructure of RM, as well as the effects of excess VAE, samples with VAE contents of 0%, 4%, and 8% at 28 days were analyzed, as shown in Figure 12.

In Figure 12a, it is observed that the RM without VAE exhibits a dense microstructure, where many gel phases are encased by needle- and rod-shaped crystalline substances, forming a compact, plate-like structure with relatively few pores in the matrix. This crystalline substance is primarily ettringite (Aft), which is not prominently visible in RM samples, and the matrix is predominantly composed of gel phases.

When 4% VAE is added, as shown in Figure 12b, the VAE interferes with the formation of C-S-H phases, exposing the needle- and rod-shaped ettringite crystals. Additionally, due to the air-entraining effect of VAE, the micro-pores within the matrix increase, explaining the reduction in compressive strength observed in VAE-modified RM. Furthermore, an interlocked composite matrix forms as ettringite crystals, unhydrated cement particles, and polymer films bond together, enhancing the flexural and tensile strength of the VAE–RM system.

In Figure 12c, when the VAE content reaches 8%, the polymer films develop more fully and distinctly. However, a greater number of needle- and rod-shaped Aft crystals are exposed, indicating reduced formation of C-S-H and increased pore size within the cement matrix. Consequently, when VAE content reaches 8%, the mechanical properties of RM exhibit varying degrees of deterioration.

## 4. Conclusions

This study modifies rubber mortar using VAE and investigates the effects of different VAE contents on the workability, mechanical properties, tensile performance, early-age crack resistance, water permeability, hydration heat, and microstructure. The main conclusions are as follows.

(1) VAE improves the fluidity of RM; however, the improvement effect decreases gradually with increasing VAE content. The fluidity is improved by 22.5% at 8% VAE content. VAE also improves the early-age crack resistance and water permeability resistance of RM.

(2) VAE is not conducive to the development of compressive strength; for every 1% VAE added, the 28 d compressive strength decreased by about 3.28%. A 4% VAE content is favorable to the flexural strength, tensile strength and ultimate tensile strain of RM, providing increases of 18.97%, 11.6% and 62.94%, respectively. However, exceeding a 4% VAE content leads to a decline in these properties.

(3) The introduction of VAE significantly extends the induction period of the hydration reaction and decreases the total hydration heat amount. This phenomenon explains the negative impact of VAE on compressive strength. Scanning electron microscopy (SEM) observations reveal that VAE forms polymer films within RM, intertwining with hydration products to create an interlocking network structure and, thereby enhancing the flexural and tensile strength of RM. However, excessive VAE will introduce too many large-pore voids, which negatively impact the mechanical strength of RM.

(4) When the VAE content is 4%, the 28-day compressive strength, flexural strength, and tensile strength of the VAE-modified RM reach 14.4 MPa, 4.83 MPa, and 1.92 MPa, respectively. The ultimate tensile strain and tensile elastic modulus are 6 GPa and 233 × 10^−6^, indicating high flexibility and ductility. In addition, the modified RM exhibits good early-age crack resistance and impermeability.

In summary, when the VAE content is 4%, VAE-modified RM exhibits superior workability, flexibility, and crack resistance. These findings provide important insights and data references for the development of new repair mortars with high ductility and crack resistance. However, this study has certain limitations. Specifically, while the macro characteristics such as the workability and mechanical properties of VAE-modified RM were extensively analyzed, the microstructural mechanisms were only explored through SEM and heat-of-hydration tests. Future research could employ a variety of microscopic analytical techniques, such as multi-point SEM sampling, X-ray diffraction (XRD), thermogravimetric analysis (TGA), Raman spectroscopy, mercury intrusion porosimetry (MIP), and Fourier-transform infrared spectroscopy (FTIR), to conduct a more comprehensive investigation of the VAE modification mechanism. This will contribute to a deeper understanding of the impact of VAE on the microstructure of RM and provide a theoretical basis for optimizing its performance.

## Figures and Tables

**Table 1:** Main chemical composition of cement (%).

CaO	SiO_2_	Al_2_O_3_	Fe_2_O_3_	SO_3_	MgO	K_2_O	Na_2_O	LOI
62.37	21.04	5.18	3.64	2.01	1.34	0.51	0.13	3.77

**Table 2:** Mortar mixture proportion.

Specimen ID.	Percentage Distribution/(g·kg^−1^)				
	Water	Cement	Sand	Rubber	VAE
VAE-0	213.48	561.80	100.08	124.64	0
VAE-2	213.48	561.80	100.08	124.64	11.24
VAE-4	213.48	561.80	100.08	124.64	22.47
VAE-6	213.48	561.80	100.08	124.64	33.71
VAE-8	213.48	561.80	100.08	124.64	44.94

**Table 3:** Test performance and sample specifications.

Performance	Sample Dimensions (mm)	Number of Samples	Age (Days)	Testing Standards
Fluidity	-	3	-	GB/T2419-2005 [26]
Compressive strength	40 × 40 × 160	3	7, 28	GB/T17671-2021 [27]
Flexural strength	40 × 40 × 160	6	7, 28	GB/T17671-2021 [27]
Tensile strength	Dumbbell shape	6	7, 28	SL352-2020 [28]
Tensile modulus	Dumbbell shape	6	7, 28	SL352-2020 [28]
Ultimate tensile strain	Dumbbell shape	6	7, 28	SL352-2020 [28]
Early-age crack resistance	600 × 600 × 63	2	2	JTG 3420-2020 [29]
Water permeability	Frustum-shaped	3	28	SL352-2020 [28]

**Table 4:** Early-age crack resistance rating criteria.

**Level**	I	II	III	IV	V
**Criteria met**	4/4	3/4	2/4	1/4	0

**Table 5:** Hydration heat test mixture proportion.

No.	Water (g)	Cement (g)	VAE (g)
VAE-0	1.900	5.000	0
VAE-2	1.863	4.902	0.098
VAE-4	1.821	4.808	0.192
VAE-8	1.759	4.630	0.370

## Data Availability

Data are contained within the article.
